# Nomogram based on MRI can preoperatively predict brain invasion in meningioma

**DOI:** 10.1007/s10143-022-01872-7

**Published:** 2022-09-30

**Authors:** Jing Zhang, Yuntai Cao, Guojin Zhang, Zhiyong Zhao, Jianqing Sun, Wenyi Li, Jialiang Ren, Tao Han, Junlin Zhou, Kuntao Chen

**Affiliations:** 1 Department of Radiology, The Fifth Affiliated Hospital of Zunyi Medical University, Zhufengdadao No.1439, Doumen District, Zhuhai, 519110 China; 2 Department of Radiology, Affiliated Hospital of Qinghai University, Xining, China; 3 Department of Radiology, Sichuan Provincial People’s Hospital, University of Electronic Science and Technology of China, Chengdu, China; 4 Department of Radiology, Lanzhou University Second Hospital, Cuiyingmen No. 82, Chengguan District, 730030 Lanzhou, People’s Republic of China; 5 Key Laboratory of Central Research Institute, United Imaging Healthcare, Shanghai, China; 6 Department of Pharmaceuticals Diagnosis, GE Healthcare, Beijing, China

**Keywords:** Meningioma, Brain, Magnetic resonance imaging

## Abstract

Predicting brain invasion preoperatively should help to guide surgical decision-making and aid the prediction of meningioma grading and prognosis. However, only a few imaging features have been identified to aid prediction. This study aimed to develop and validate an MRI-based nomogram to predict brain invasion by meningioma. In this retrospective study, 658 patients were examined via routine MRI before undergoing surgery and were diagnosed with meningioma by histopathology. Least absolute shrinkage and selection operator (LASSO) regularization was used to determine the optimal combination of clinical characteristics and MRI features for predicting brain invasion by meningiomas. Logistic regression and receiver operating characteristic (ROC) curve analyses were used to determine the discriminatory ability. Furthermore, a nomogram was constructed using the optimal MRI features, and decision curve analysis was used to validate the clinical usefulness of the nomogram. Eighty-one patients with brain invasion and 577 patients without invasion were enrolled. According to LASSO regularization, tumour shape, tumour boundary, peritumoral oedema, and maximum diameter were independent predictors of brain invasion. The model showed good discriminatory ability for predicting brain invasion in meningiomas, with an AUC of 0.905 (95% CI, 0.871–0.940) vs 0.898 (95% CI, 0.849–0.947) and sensitivity of 93.0% vs 92.6% in the training vs validation cohorts. Our predictive model based on MRI features showed good performance and high sensitivity for predicting the risk of brain invasion in meningiomas and can be applied in the clinical setting.

**Supplementary Information:**

The online version contains supplementary material available at 10.1007/s10143-022-01872-7.

## Introduction

Meningiomas are the most common primary intracranial tumours in adults, accounting for approximately 36.7% of all intracranial tumours [1]. According to the 2016 edition of the World Health Organization (WHO) classification of central nervous system tumours [2], the microscopic examination of brain invasion has become a stand-alone criterion for the differentiation of grade II atypical meningioma. This new criterion has the highest clinical relevance. Brain invasion by meningioma is not only associated with surgical decision-making but is also independently associated with recurrence [3, 4] and a poor prognosis [5]; it is therefore indirectly related to adjuvant therapy and to inclusion in clinical trials. In addition, brain invasion is a risk factor for preoperative seizures and postoperative haemorrhage, which has gained distinct changes in clinical behaviour [6, 7]. For the abovementioned reasons, brain invasion has important clinical significance and is receiving the increased attention from clinicians [8]. Therefore, it is necessary to accurately predict brain invasion by meningiomas before surgery.

At present, the only standard for diagnosing brain invasion in meningiomas is histopathological examination. However, extensive brain tissue sampling at the tumour-brain interface during surgical resection is difficult, and standardized surgical sampling and neuropathological analysis are still inconsistent. Therefore, brain invasion may not be detected by histopathology [9, 10]. Some authors reported that 85% of the samples were “unassessable” pathologically [6]. Imaging studies of brain invasion can analyze the entire tumour compared to focusing on local fine structures. According to the guidelines of the European Association of Neuro-Oncology, MRI is the main method for the provisional diagnosis of meningioma. Several studies have explored the correlation between brain invasion and imaging features [6, 11, 12], such as peritumoral oedema, heterogeneous contrast enhancement, and irregular tumour shape, which have been identified as predictors of brain invasion [6]. However, only a few studies have explored reliable imaging-related predictors of brain invasion, and their results remain controversial [5]. Although these studies have found a correlation between imaging features and brain invasion, few studies have developed a predictive model and independently verified these results to confirm their accuracy and reliability.

Thus, in this study, we developed and validated a predictive model to investigate the potential association between MRI features and brain invasion. It is hypothesized that a predictive model may offer a more accurate non-invasive preoperative prediction of brain invasion in meningiomas. Therefore, the aim of our study was to (1) select MRI features that are correlated with brain invasion in meningiomas, (2) combine these MRI features to build a predictive model, and (3) establish a nomogram to predict brain invasion in patients with meningioma based on MRI.

## Materials and methods

### Patient cohort

This was a retrospective study. Ethical approval was obtained from the Institutional Review Board of Lanzhou University Second Hospital, and the requirement for informed patient consent was waived. All patients with meningiomas who underwent surgery were enrolled according to the inclusion and exclusion criteria. The inclusion criteria were as follows: (1) a diagnosis of meningioma and clear grading by histology according to the 2016 edition of the WHO classification of central nervous system tumours; and (2) available MR images, including T1, T1C, and T2 sequences, taken within 1 week before the surgical resection. The exclusion criteria were as follows: (1) patients without a clear histological grading, (2) patient images with artefacts and poor quality that impacted assessment, (3) patients with incomplete MRI sequences, and (4) patients with surgeon-suspected brain invasion.

With the aid of a microscope, tumour resection was performed for all patients, and brain invasion was diagnosed according to the pathological records [13]. Because of the limitations of extensive brain tissue sampling, some cases may not have been determined as brain invasion. The samples (excluding poor quality imaging, brain invasion, etc.) were further assessed for brain invasion by the surgeon [14]. Surgical records described obvious adhesions at the tumour-brain interface, and these were recorded as brain invasion. Thus, 16 patients with suspected brain invasion were excluded.

**Fig. 1:**
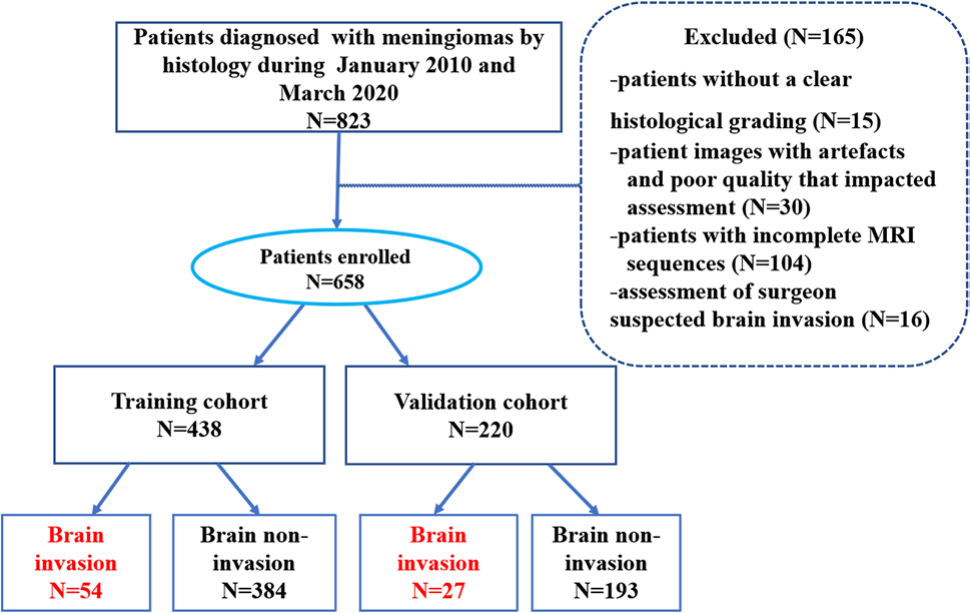
Inclusion and exclusion criteria

A total of 658 patients at Lanzhou University Second Hospital were enrolled from January 2010 to March 2020. After surgeon evaluation, 81 patients (training cohort: *n* = 54; validation cohort: *n* = 27) were enrolled in the invasion group, while 577 patients (training cohort: *n* = 384; validation cohort: *n* = 193) were enrolled in the non-invasion group. Subsequently, the patients were randomly divided into training (*n* = 438, 86 male and 352 female, mean age 52.24 ± 9.97 years) and validation (*n* = 220, 51 male and 169 female, mean age 51.28 ± 10.23 years) cohorts in a 7:3 ratio. The patient recruitment flowchart is shown in Fig. 1. Data concerning the following two conventional clinical variables, age and sex, were obtained from the electronic medical records.

### MRI parameters

MRI scans were performed at our institution with a 1.5-T scanner (Siemens Magnetom Aera) and 3.0-T scanners (Philips Achieva; Siemens Verio). All MRI examinations included T2-weighted, T1-weighted, and T1-weighted sequences after the administration of a gadolinium-based contrast agent with fat suppression. The detailed MRI parameters are provided in Table S1.

### Imaging analysis

**Fig. 2:**
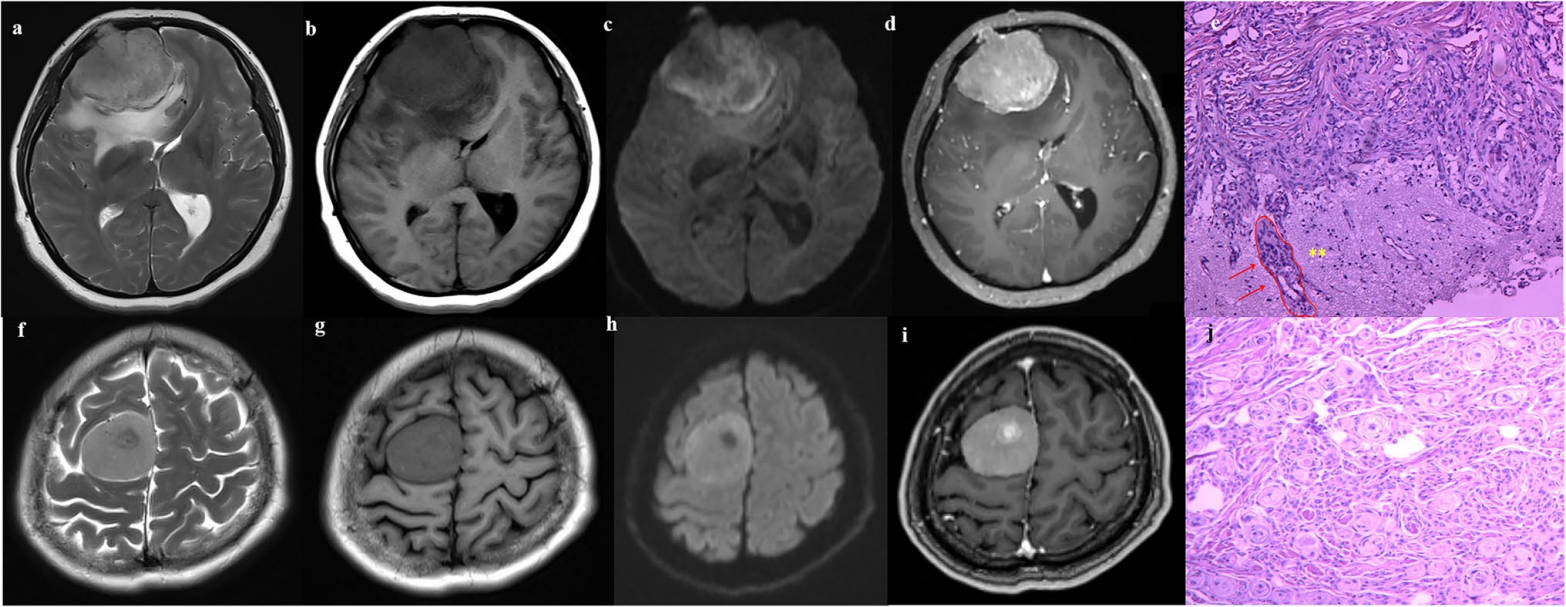
Examples of different MRI features of meningiomas in the invasion and non-invasion groups. **A**–**D** A 51-year-old female with brain invasion in transitional meningioma. The tumour is irregular in shape and grows across the leaves. On MRI, the tumour shows uneven slight high signal on T2WI, low signal on T1WI, and uneven high signal on DWI. Peritumoral oedema is defined as 34–67%. T1 enhancement manifests as significantly reinforcement and bone invasion. In **E**, transitional meningioma, tumour shows finger-like expansions (red arrows) in the surrounding brain tissue. (haematoxylin and eosin staining × 200; ** = brain tissue). **F**–**I** A 49-year-old female with brain non-invasion in transitional meningioma. The tumour is round in shape. On MRI, the tumour shows slight high signal on T2WI, slight low signal on T1WI, and uneven high signal on DWI. A complete cerebrospinal fluid cleft is visible, and no peritumoral oedema. T1 enhancement manifests as significantly reinforcement. **J** The epithelial-like cells are arranged in nests, the cytoplasm is acidophilic, the nucleus is round, and some areas form a whirlpool structure. (hematoxylin and eosin staining × 200)

Two neuroradiologists (readers 1 and 2, with 12 and 15 years of experience in brain MRI interpretation, respectively) independently evaluated 658 MRI data sets and were blinded to the diagnosis and results of each other. If there was any disagreement, a unanimous decision was reached after discussion. In terms of morphological characteristics, a total of nine MRI features was assessed according to literature reports [15, 16] and previous research experience, as shown in Table 1. The tumour maximum diameter was measured on T1C images, and the average of the maximum diameters provided by both neuroradiologists was then calculated, and the dural tail was not included in the measurement. Enhanced features including uniform enhancement (nonenhancing portions due to the presence of cysts, necrosis, haemorrhage, or inadequate blood supply) or uneven enhancement (complete filling of contrast without any intervening structure inside the tumour) was assessed on T1C images. Peritumoral oedema was evaluated on T2 images according to the standardized visually accessible Rembrandt Images (VASARI; https://wiki.nci.nih.gov/display/CIP/VASARI) feature set. Peritumoural oedema should be greater in signal than nonenhancing tumour and somewhat lower in signal than cerebrospinal fluid. Percentage represents the proportion of peritumoural oedema in the entire abnormality, which included the entire tumour and oedema component. Bone invasion assessments were performed by pathology and surgeon assessment intraoperatively [14, 17]. Sinus invasion was evaluated by an intraoperative neurosurgeon as a diagnostic standard [18]. We calculated the interclass correlation coefficients in order to evaluate the reproducibility of MRI judged by the two observers, and values greater than 0.75 indicated good agreement. Examples of different MRI features of meningiomas in the invasion and non-invasion groups are shown in Fig. 2.

**Table 1:** Clinical factors and MRI features of brain invasion and non-invasion groups in meningioma (mean ± SD or no, %)

Characteristics	Brain invasion (*n* = 81)	Non invasion (*n* = 577)	Univariate analysis (*p* value)
Clinical factors			
Age (years)	52.5 ± 10.5	52.2 ± 10.70	0.833
Sex			< 0.001*
Female	48 (59.3%)	474(82.1%)	
Male	33 (40.7%)	103 (17.9%)	
Imaging features			
Maximum diameter (mm)	52.44 ± 15.05	35.88 ± 14.28	< 0.001*
Tumour shape			< 0.001*
Circular or quasi- circular	23 (28.4%)	447(77.5%)	
Irregular	58 (71.6%)	130 (22.5%)	
Tumour boundary			< 0.001*
Clear	63 (77.8%)	548(95%)	
Blur	18 (22.2%)	29(5%)	
Dural tail sign			0.341
Yes	41(50.6%)	257(44.5%)	
None	40(49.4%)	320(55.5%)	
Peritumoural oedema			< 0.001*
1 Uncertain	0(0%)	0(0%)	
2 None (0%)	6(7.4%)	329(57.0%)	
3 ≤ 5%	9(11.1%)	71(12.3%)	
4 6–33%	20(24.7%)	84(14.6%)	
5 34–67%	22(27.2%)	55(14.0%)	
6 68–95%	23(28.4%)	33(6.7%)	
7 > 95%	1(1.2%)	5(0.9%)	
MRI signal			
T2WI			0.115
Slightly high signal	34 (42.0%)	191 (33.1%)	
Iso signal	22 (27.2%)	221 (43.2%)	
Mixed signal	25 (30.8%)	165 (28.6%)	
Enhanced features			< 0.001*
Uniform	19 (23.5%)	418(72.4%)	
Uneven enhancement	62 (76.5%)	159(27.6%)	
Bone invasion			0.124
Yes	36(44.4%)	195(33.8%)	
No	45(55.6%)	382(66.2%)	
Sinus invasion			0.381
Yes	24(29.6%)	158(27.4%)	
No	57(70.4%)	419(72.6%)	

Among peritumoural oedema, percentage represents the proportion of peritumoural oedema in the entire abnormality, and the entire abnormality may be comprised of the entire tumour and oedema component. A Student’s *t*-test was used to compare the difference in age and maximum diameter, while the chi-square test was used to compare the difference in other features. *P < 0.05

*SD* standard deviation

### Nomogram development and validation

Based on the MRI features selected by LASSO, multivariate logistic regression analysis was used to build a predictive model for predicting the risk of brain invasion. Thereafter, using the coefficients of the multivariate logistic regression, a nomogram combining correlated MRI features was developed in the training cohort and validated in the validation cohort. This method provides a more understandable and convenient tool for patients and clinicians.

The calculation formula for the nomogram:

Nomogram score =  − 5.4709 + 0.511 × Tumour shape + 0.256 × Enhanced features + 0.373 × Oedema + 0.026 × Maximum diameter.

The discriminatory ability of the nomogram was assessed by calibration curves for the training and validation cohorts, which showed agreement between the observed outcomes and predicted risk of brain invasion. Decision curve analysis was used to quantify the net benefits at different threshold probabilities in order to assess the clinical usefulness of the nomogram [19].

### Statistical analysis

All statistical analyses were performed using the R software (version 3.6.0; http://www.Rproject.org). Two-sided *P*-values of < 0.05 were considered statistically significant. Descriptive statistics for continuous variables are expressed as mean ± standard deviation, and discrete variables are expressed as percentages. In the training and validation cohorts, the chi-square test and Student *t*-test were used to compare the differences in the clinical characteristics and MRI features between the invasion and non-invasion groups. Generally, two-sided *P* values < 0.05 were considered statistically significant. The least absolute shrinkage and selection operator (LASSO) regression was used to select highly related factors of brain invasion in order to reduce the risk of overfitting. The AUC was used to evaluate the discriminatory ability of the predictive model [20]. The larger the AUC value, the better the discriminatory ability of the predictive model. Generally, AUC > 0.75 was considered to have excellent discrimination [21].

## Results

### Clinical characteristics and MRI features

**Fig. 3:**
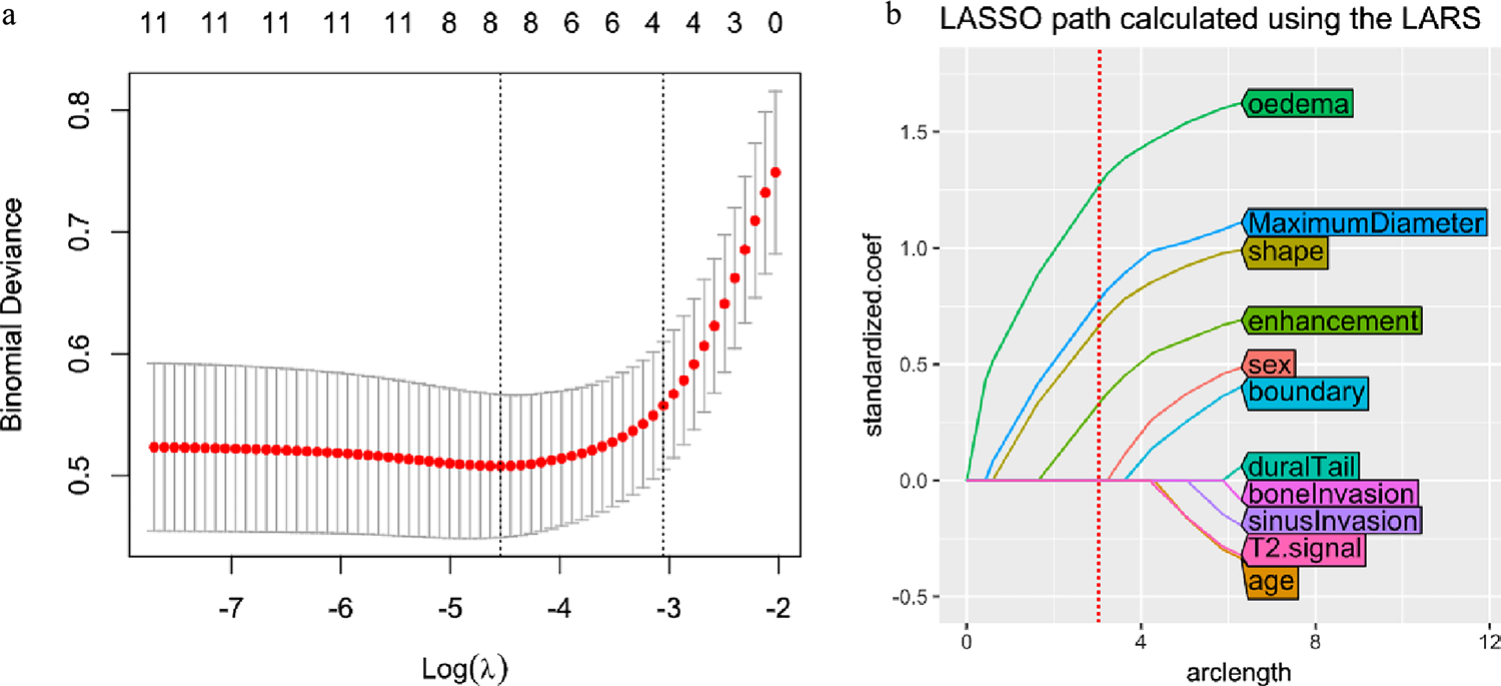
Parameter selection by the LASSO regression procedure. **A** A tenfold cross-validation was used in the LASSO regression procedure. Binomial deviance was delineated versus log (lambda). The dotted vertical lines were drawn at the optimal values by utilizing the minimum criteria (left dotted line) and the 1-standard error criterion (right dotted line). **B** The LASSO coefficient profiles of the 11 variables. A coefficient profile plot was produced in connection with the log (lambda) sequence. The dotted vertical lines were drawn at the optimal values the 1-standard error criterion (red line). In the study, as penalization increased (to the right of the figure), parameters were chosen according to the optimal values, where only four coefficients related to the optimal lambda were non-zero. The best predicting model was constructed using the four remaining parameters. LASSO, least absolute shrinkage and selection operator

The clinical characteristics and MRI features of the patients are shown in Table 1. According to the Student *t*-test and chi-square test, six features (including sex, maximum diameter, tumour shape, tumour boundary, peritumoral oedema, and enhanced features) were significantly different between the invasion and non-invasion groups after univariate analysis (*P* < 0.05). These six variables were further reduced to four MRI features regarded as optimal predictors by LASSO regularization; these predictors had non-zero coefficients. The LASSO algorithm prefers estimation of sparse coefficients using a linear model with a L1 regularization penalty term, resulting in fewer selected features with non-zero coefficient for later modelling procedure. Besides, cross-validation method was used to determine the best regularization parameter in LASSO algorithm, called LassoCV. In this study, the coefficient of L1 term (lamda) was chosen as 0.01065. These variables were tumour shape (coefficient, e0.511), enhanced features (coefficient, e0.256), peritumoral oedema (coefficient, e0.373), and maximum diameter (coefficient, e0.026). Ten-fold cross-validation was used for the tuning parameter (lambda) selection, as shown in Fig. 3.

### Model development and validation

**Fig. 4:**
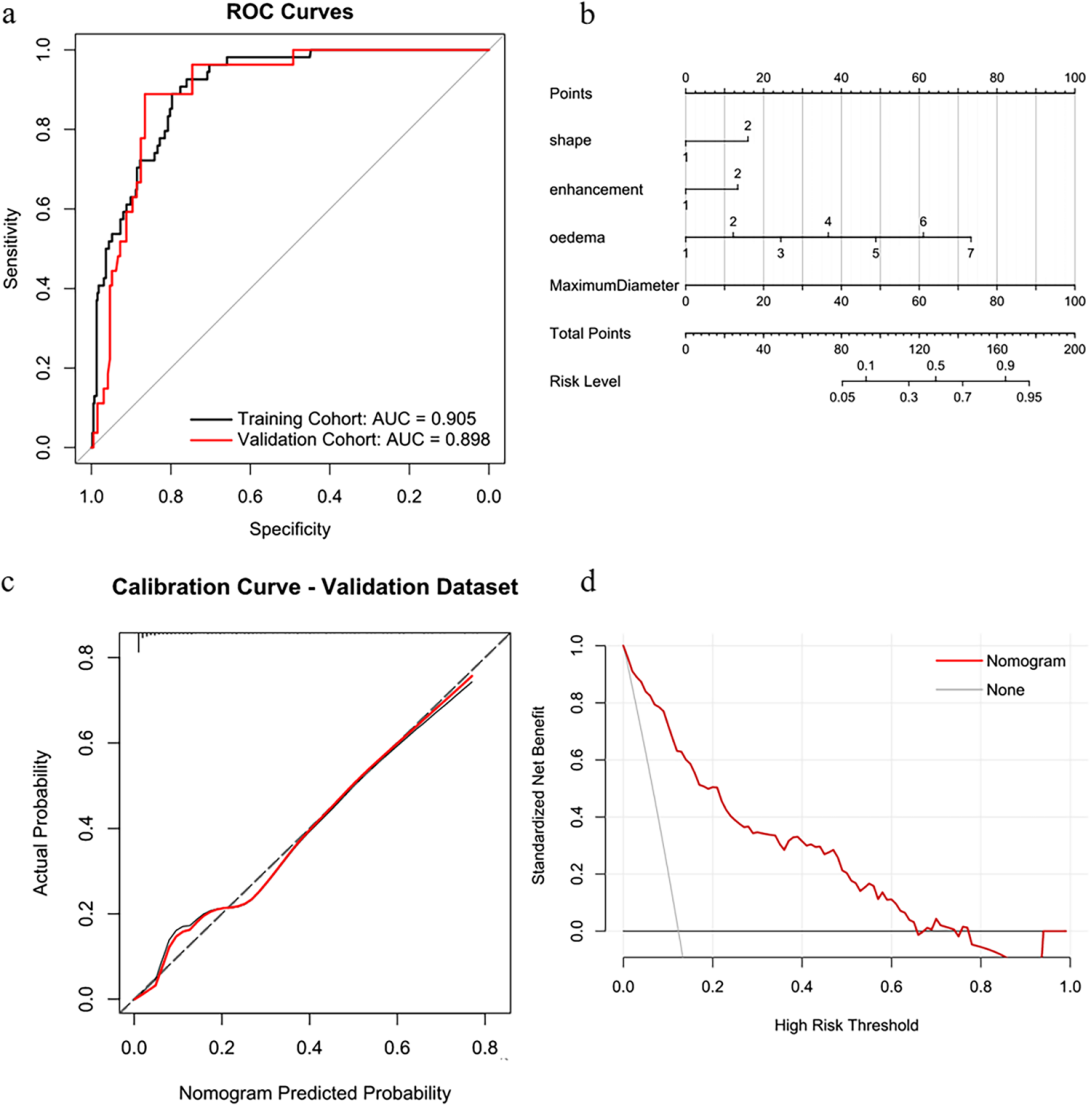
Establishment and performance of the model. **A** The receiver operating characteristic (ROC) curves of the predictive model. The model demonstrated good discriminating ability, with an AUC of 0.905 in the training cohort and an AUC of 0.898 in the validation cohort. **B** The predictive model was conducted to develop a nomogram. **C** Calibration curves of the nomogram for the training cohort. The *x*-axis represents the probability of brain invasion measured using the model, and the *y*-axis represents the actual rate of brain invasion. The red represents the discrimination ability of the nomogram, while the diagonal dotted line represents an ideal evaluation by a perfect model. A closer fit to the diagonal dotted line represents a better evaluation. **D** Decision curve analysis for the predictive model. The *x*-axis shows the threshold probability, and the *y*-axis measures the net benefit. The gray line represents all patients with brain invasion, while the black line represents all patients without brain invasion. The red line represents the predictive model

Based on the selected MRI features, we built a predictive model using multivariate logistic regression analysis to predict the risk of brain invasion. The predictive model demonstrated good performance and defined the nomogram, resulting in an AUC of 0.905 (95% CI, 0.871–0.940) vs 0.898 (95% CI, 0.849–0.947), a sensitivity of 93.0% (95% CI, 83.3–100%) vs 92.6% (95% CI, 81.5–100%), accuracy of 79.7% (95% CI, 71.2–86.3%) vs 86.4% (95% CI, 74.5–90.9%), and specificity of 77.8% (95% CI, 67.9–86.5%) vs 86.0% (95% CI, 71.5–91.1%) for brain invasion prediction in the training vs validation cohorts, as shown in Table 2 and Fig. 4a.

**Table 2:** Performance of the predictive model

Cohort	AUC	ACC	SEN	SPE
Training cohort	0.905 (0.871–0.940)	0.797 (0.712–0.863)	0.930 (0.833–1.000)	0.778 (0.679–0.865)
Validation cohort	0.898 (0.849–0.947)	0.864 (0.745–0.909)	0.926 (0.815–1.000)	0.860 (0.715–0.911)

*AUC* area under receiver operating characteristic curve, *ACC* balanced accuracy, *SEN* sensitivity, *SPE* specificity, *NPV* negative predictive value, *PPV* positive predictive value

### Nomogram performance assessment

The predictive model showed good performance and defined the nomogram (Fig. 4b). A calibration curve was applied to measure the consistency between the actual brain invasion outcomes and the probability of brain invasion predicted by the model. As shown in Fig. 4c, the actual brain invasion outcomes were consistent with the predicted probability of brain invasion in both the training and validation cohorts (all *P* > 0.05). The decision curve then assessed the discriminatory ability of the model based on clinical applications. The model provided a net benefit in the decision curve analysis over the brain invasion scheme or non-invasion scheme at a threshold probability of > 10% (Fig. 4d).

## Discussion

In this study, we developed and validated a nomogram based on MRI features, including tumour shape, enhanced features, peritumoral oedema, and maximum diameter, to predict brain invasion in patients with meningioma. The nomogram showed good discriminatory ability (AUC: 0.905 vs 0.898) and high sensitivity (93.0% vs 92.6%) in both the training and validation cohorts. The performance of this nomogram was validated via discrimination and calibration curves in an independent validation cohort and should enable more accurate prediction of brain invasion.

In this study, the incidence of brain invasion (12.3%) is consistent with other reports (4–19%) amongst tumours of all WHO grades [13, 22]. Among clinical characteristics, sex was significantly different between the brain invasion and non-invasion groups after univariate analysis. Male patients were prone to brain invasion, which is in agreement with previously published reports. Recent studies have found that there are a higher proportion of males with invasive meningiomas than with non-invasive meningiomas [6, 23, 24]. Moreover, the invasive patterns were different. Brain invasion demonstrated a predominantly cluster-like infiltrative growth in women, while the pattern showed a mostly finger-like growth in men [5]. In the analysis of continuous variables, there was no significant difference in mean age between the invasion and non-invasion groups, which is consistent with other reports. Brokinkel et al. [5] conducted a systematic review of the literature, analyzed 15 studies, and concluded that the incidence of brain invasion was generally not correlated with patient age.

MRI is the main method for the diagnosis of meningioma and may serve as a non-invasive approach for the visual assessment of brain tissue around meningiomas. Thus, we explored the relationship between MRI features and brain invasion in meningiomas. After LASSO regularization analysis, four MRI features, including tumour shape, enhanced features, peritumoral oedema, and maximum diameter, were strongly correlated with brain invasion in meningiomas and could be used as optimal independent risk predictors. Compared with non-invasive meningioma (22.5%), the tumours in the brain invasion group were more irregular (71.6%), which is consistent with the findings of some previous studies. Adeli et al. [6] found that tumour shape was significantly different between the brain invasion and non-invasion groups on univariable analyses, as noted in our results. Previous studies have shown a correlation between atypical meningiomas and irregular margins [15, 25, 26]. Our result demonstrated that the irregular tumour shape corresponded to an “invasive” phenotype [27] and tended to grow in all directions at different rates. However, Ong et al. reported that brain invasion was not associated with tumour contour [28]. This discrepancy could be attributed to the sample size. The heterogeneous enhancement pattern of meningiomas mostly indicates cyst formation, necrosis, or haemorrhage. In accordance with previous studies, uneven contrast enhancement was correlated with high-grade histology [29]. In our study, enhanced features were significantly different between the two groups. Similar findings [6] were revealed when analyzing correlations of uneven enhancement with brain invasive growth or other grading criteria separately.

In addition, peritumoral oedema was significantly different between the brain invasion and non-invasion groups. After LASSO analysis, there was a strong correlation between peritumoral oedema and brain invasion, which was consistent with the results of previous studies [6, 28, 30]. Several authors have reported a strong correlation between an invasive growth pattern and peritumoral oedema, and Mantle et al. found a 20% increase in brain invasion incidence for each centimetre of oedema [11], while Gill et al. reported that every 1-cm^3^ increase in peritumoral oedema was associated with a 10% increase in the odds of brain invasion [30]. Peritumoral oedema is often associated with the loss of a clean arachnoid dissection plane at the tumour-brain interface, decreased vascular endothelial growth factor (VEGF) expression, and impaired pial blood supply to the meningioma [31, 32]. The maximum diameter was statistically significant for identifying brain invasion; this finding was different from the results of previous studies. The results indicated that the larger the maximum diameter of the meningioma, the greater the probability of brain invasion. Adeli et al. found that brain invasion was not associated with a larger tumour volume [6]. The main reason for this difference is that, on one hand, we measured the maximum diameter at the largest section of the tumour and could not reflect the overall size of the tumour, while previous studies measured the tumour volume. The difference between maximum diameter and tumour volume may have a certain deviation. On the other hand, there are few imaging studies on brain invasion in meningioma, and the criteria for including imaging features differed between studies. In the future, further research with larger sample sizes from different centres is needed.

To conclusively identify the MRI features associated with brain invasion, 11 candidate variables (including two clinical characteristics and nine MRI features) were reduced to four potential predictors based on LASSO regularization. LASSO regularization avoids overfitting and allows the analysis of multiple characteristics of cohorts with relatively small sample sizes [33]. Based on these four predictors, both the final model and independent internal validation demonstrated good discriminatory ability. Moreover, compared to the model, combining the four predictive factors of the nomogram demonstrated good predictive performance. This individualized nomogram should contribute to the preoperative prediction of brain invasion in meningiomas for both radiologists and clinicians. The results of the nomogram were more beneficial than those of the predictive model and could be used in clinical applications for meningioma patients who undergo MRI.

This study has a few limitations. First, it had a retrospective study design, and our results may have been affected by selection bias. Second, this was a single-centre study, and a multicenter study is needed to validate the robustness and generalizability of the model. Third, MRI features were judged by subjective and qualitative evaluations, which could be non-specific. Moreover, after assessment of surgeon, 16 patients with suspected brain invasion were excluded, which may cause data deviation.

## Conclusion

The preoperative identification of brain invasion should facilitate improvements in clinical decision-making, prediction of meningioma grading, and prognosis. Four MRI features, including tumour shape, enhanced features, peritumoral oedema, and maximum diameter, demonstrated high correlations with brain invasion. The model incorporating these four MRI features showed excellent performance and high sensitivity in predicting brain invasion and can be used in patients with meningioma.

## Supplementary Information

Below is the link to the electronic supplementary material.

Supplementary file1 (DOC 33 KB)

## Data Availability

Not applicable
