# Supplementary material for: Nomogram based on MRI can preoperatively predict brain invasion in meningioma
Source: Neurosurg Rev. 2022 Sep 30;45(6):3729–37. doi: 10.1007/s10143-022-01872-7 (PMC9663361; doi:10.1007/s10143-022-01872-7)
Supplement: Supplementary file 1 — Supplementary file1 (DOC 33 KB) [file 10143_2022_1872_MOESM1_ESM.doc]

**Supplementary Material**

**List**

**1. Supplementary TableS1**

**Supplementary TableS1**

**Table S1** MRI parameters of the scanning sequences

| Scanner | Patients No | Sequence | TR  (ms) | TE  (ms) | Matrix | Slice  Thickness (mm) | Slice Gap  (mm) | Slices | Flip  Angle | Acquisition Time |
| --- | --- | --- | --- | --- | --- | --- | --- | --- | --- | --- |
| Siemens Verio | 201 | T1C  T1 | 1900  1800 | 2.9  9 | 286 x256  320x237 | 5.0  5.5 | 1.65  1.65 | 20  20 | 150  150 | 1min8sec  1min8sec |
|  |  | T2 | 4500 | 91 | 336 x448 | 5.5 | 1.65 | 18 | 150 | 59sec |
| Philips Achieva | 20 | T1C  T1 | 450  450 | 15  15 | 256x256  256x256 | 6.0  6.0 | 1.17  1.17 | 18  18 | 69  69 | 1min2sec  1min2sec |
|  |  | T2 | 3000 | 100 | 512x512 | 6.0 | 1.17 | 18 | 100 | 1min21sec |
| Siemens Magnetom Aera | 22 | T1C  T1 | 2000  2000 | 106  106 | 320x224  320x224 | 6.0  6.0 | 0.9  0.9 | 20  20 | 150  146 | 48s  48s |
|  |  | T2 | 2200 | 106 | 320x320 | 6.0 | 0.9 | 20 | 146 | 53sec |

Abbreviations: T1C, Contrast enhancement T1-weight imaging; T2, T2-weight imaging; TR, repetition time; TE, echo time.
